# Development of digital biomarkers for resting tremor and bradykinesia using a wrist-worn wearable device

**DOI:** 10.1038/s41746-019-0217-7

**Published:** 2020-01-15

**Authors:** Nikhil Mahadevan, Charmaine Demanuele, Hao Zhang, Dmitri Volfson, Bryan Ho, Michael Kelley Erb, Shyamal Patel

**Affiliations:** 1 Pfizer, Inc., Cambridge, MA 02139 USA; 2 Tufts Medical Center, Boston, MA 02111 USA

**Keywords:** Biomedical engineering, Biomarkers

## Abstract

Objective assessment of Parkinson’s disease symptoms during daily life can help improve disease management and accelerate the development of new therapies. However, many current approaches require the use of multiple devices, or performance of prescribed motor activities, which makes them ill-suited for free-living conditions. Furthermore, there is a lack of open methods that have demonstrated both criterion and discriminative validity for continuous objective assessment of motor symptoms in this population. Hence, there is a need for systems that can reduce patient burden by using a minimal sensor setup while continuously capturing clinically meaningful measures of motor symptom severity under free-living conditions. We propose a method that sequentially processes epochs of raw sensor data from a single wrist-worn accelerometer by using heuristic and machine learning models in a hierarchical framework to provide continuous monitoring of tremor and bradykinesia. Results show that sensor derived continuous measures of resting tremor and bradykinesia achieve good to strong agreement with clinical assessment of symptom severity and are able to discriminate between treatment-related changes in motor states.

## Introduction

Parkinson’s disease (PD) is one of the most common neurodegenerative disorders affecting approximately 10 million people worldwide.^1–3^ The loss of dopaminergic neurons in the substantia nigra region of the midbrain, which is critical for motor control, is a primary contributor to the pathophysiology of PD.^3–5^ Tremor, bradykinesia (i.e., slowness of movement), postural instability and rigidity (i.e., stiffness and resistance to passive movement) are the cardinal motor symptoms of PD.^1^ In addition, patients also experience non-motor symptoms like dysarthria, hyposmia, sleep disorders, and cognitive impairments. Collectively, these symptoms have a significant impact on the functional ability of patients and their overall quality of life. In early stages of the disease, dopamine replacement therapy, such as levodopa (L-Dopa), is highly effective at controlling motor symptoms. However, as the disease progresses, patients often develop complications like dyskinesias and motor fluctuations, which reduces the efficacy of levodopa-based treatments.^6^ Despite many advances in the development of therapies for managing PD symptoms, there are currently no available therapies that slow disease progression or address the wide range of symptoms in patients with more advanced PD.^7^

The use of subjective, episodic, and insensitive clinical assessment tools,^8^ which provide sparse data and poor ecological validity, can be an impediment to the development of new therapies. The current standard for clinical assessment of PD is the Movement Disorder Society’s Unified Parkinson’s Disease Rating Scale (MDS-UPDRS).^9^ Clinical assessments performed using the MDS-UPDRS are time-consuming, require the presence of a trained clinician, are inherently subjective and lack the necessary resolution to track fine grained changes (MDS-UPDRS items are rated on an ordinal scale of 0–4). A home diary^10^ completed for a few days preceding clinic visits by the patient or caregiver is another instrument that is commonly used in clinical trials for evaluating treatment efficacy based on a report of motor symptoms experienced outside the clinic. However, issues such as lack of compliance, recall bias and diary fatigue limit the accuracy of information that can be collected with this approach.^11^ The limitations of these tools contribute to the need for large sample sizes and long durations of clinical trials for new therapies, and increase the risk of failures.^12^

Advances in sensing technology have fostered the development of new methods for objective measurement of PD motor symptoms.^13^ Early efforts^14^ were focused on the development and validation of methods with data collected using multiple sensing modalities (e.g., accelerometers,^14^ gyroscopes,^15^ and electromyography^16^) often from multiple body locations during the performance of scripted motor tasks (e.g., tasks from the MDS-UPDRS) under supervision in a clinic or lab. These investigations demonstrated the feasibility of extracting clinically meaningful information from data collected using wearable sensors in the clinic. More recently, smartphone-based tools, which are easier to scale and deploy compared to wearable devices,^17–19^ are making it easier to perform frequent assessments outside the clinic. However, assessments performed using smartphones are still episodic in nature and the quality and quantity of information will be dependent on the compliance and motivation of patients. Therefore, there is significant interest in developing systems that can continuously and unobtrusively monitor motor symptoms during daily life.^20^

In order to deploy wearable sensors in a real-world setting, it is essential to develop and validate methods that are able to extract clinically meaningful information from data collected during unstructured activities typically performed under free-living conditions while relying on a minimal number of devices.^21^ In addition, the choice of sensing modality, which has a significant impact on usability of the system, also needs to be considered.^22^ For example,^15^ while gyroscopes are excellent at capturing the motion dynamics associated with tremor and other movements that involve a rotational component, power consumption of gyroscopes is typically at least an order of magnitude higher than accelerometers.^23^ As a result, devices with gyroscopes would need to be frequently recharged resulting in increased patient burden or have to be very bulky and hence have poor wearability because of a large battery.

Here, we present the development and validation of a method for continuous, objective assessment of resting tremor and bradykinesia based on data from a single wrist-worn accelerometer. The proposed method follows a hierarchical paradigm by first determining activity periods of interest (i.e., context) and then applying context specific processing steps to detect the presence of motor symptoms and derive objective measures of their severity. We demonstrate criterion validity of the system by showing that sensor-derived measures are significantly associated with corresponding clinical ratings provided by a trained examiner (“live-rater”) during in-clinic visits. In addition, we show that sensor-derived measures are significantly different between self-reported motor states (confirmed before and after medication), which demonstrates discriminative validity of the proposed system. Lastly, utility was confirmed by a marked patient preference for the wrist location and an adhesive patch form factor as well as a willingness to wear the device during daily life.

### Related work

A survey of prior work focused on development and validation of methods aimed at monitoring tremor and bradykinesia during free-living activities can be found in Supplementary Table 1. Many of these systems use multiple sensing devices^24–26^ and modalities^27–30^ to measure a number of motor symptoms that are present in PD. For example, Zwartjes et al.^29^ proposed a hierarchical framework for context specific assessment of tremor and bradykinesia using inertial sensors (accelerometer and gyroscope) located at four locations on the body. This system achieved high accuracy for activity classification (~99%), showed good agreement with clinical ratings and was responsive to changes in treatment settings (DBS “on”, “intermediate (80% of optimal settings)”, and “off”). A multi-modal system proposed by Roy et al.^28^ uses accelerometer and surface EMG data recorded from four wearable devices placed on the limbs for monitoring tremor and dyskinesia during unconstrained activity. The system achieved high (>90%) sensitivity and specificity for detection of symptoms as well as severity assessment and results show that use of EMG contributed to improved accuracy for tremor detection (~10% improvement). Systems like SENSE-PARK^31^ and PERFORM^30^ have been designed for a comprehensive assessment of a broad range of PD motor and non-motor symptoms during daily life. In addition to multiple wearable devices, they include peripheral tools like a balance board and touch screen computer to perform prescribed tests and log information about food intake, medication timings and self-assessment of symptoms. While these systems have the ability to perform continuous assessment of motor symptoms, the need for multiple device locations and technical complexity (e.g., use of EMG) are challenges that would need to be addressed before they can be deployed at scale.

In contrast, commercially available systems like Kinesia^32^ and Personal KinetiGraph (PKG)^33^ aim to minimize patient burden by reducing the number of devices for monitoring patients under free-living conditions. Using multi-modal (accelerometer and gyroscope) data recorded from devices located on the wrist and ankle of the most affected side, Pulliam et al.^27^ were able to achieve good accuracy (AUC > 0.8) for detection of tremor, bradykinesia, and dyskinesia as well as ability to differentiate (*p* < 0.01) between treatment states (ON and OFF). The PKG is a wrist-worn device for continuous at-home monitoring, which implements heuristic algorithms that process raw accelerometer data to generate a score for motor symptoms (e.g., dyskinesia and bradykinesia^34^). Measures derived using the PKG system from at home recordings have demonstrated criterion and discriminative validity,^35^ and researchers evaluating the clinical utility of these systems are finding that it could help improve disease management and improve outcomes.^36–38^ However, while the original developers of these commercially available systems have worked on clinically validating the algorithms, they are proprietary and therefore not available for others to reproduce, validate and improve. In fact, the lack of open analytical methods^20^ was one of the challenges highlighted by the MDS Taskforce on Technology.

While these efforts have certainly advanced the field, there are still significant gaps that need to be addressed.^39^ Several of these approaches rely on the use of multiple devices across different body locations with the aim of characterizing a range of motor symptoms and quality of life (e.g., gait, tremor, bradykinesia, and sleep). In addition, while most approaches demonstrate good detection accuracy for clinical features (e.g., tremor periods and freezing of gait events), there is a lack of consideration for demonstrating both criterion (i.e., association between sensor measures and clinical ratings) and discriminative validity (i.e., ability of sensor measures to discriminate between different disease states) for methods aimed at monitoring motor symptoms during free living conditions. Furthermore, there needs to be an emphasis on formal testing of the utility and acceptability^40^ of these systems as patients might be required to wear the device(s) for days or weeks at a time.

## Results

**Fig. 1:**
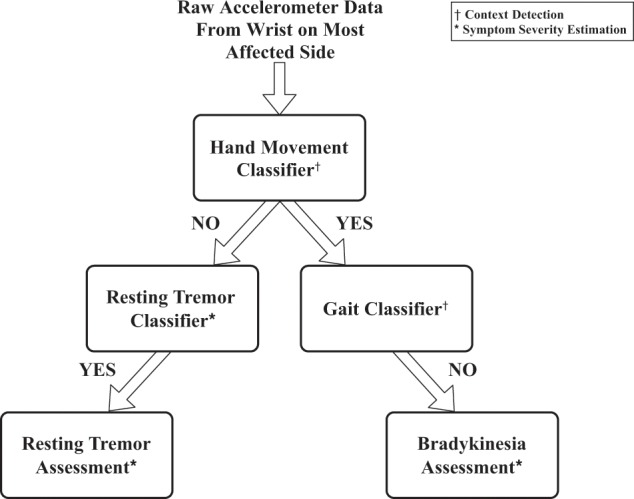
Flow chart illustrating the hierarchical approach for detection and assessment of resting tremor and hand bradykinesia using accelerometer data from a wrist-worn device. This approach utilizes context detection (hand movement and gait) to identify periods of interest from raw sensor data and subsequently performs detection and assessment of motor symptoms (tremor and bradykinesia). Tremor is assessed when the hand is at rest and bradykinesia is assessed during periods of hand movement that are not due to gait.

As illustrated in Fig. 1, our analysis approach consists of two steps: (1) context detection and, (2) symptom severity estimation. The context detection step includes detection of hand movement followed by detection of gait (if hand movement was detected). The symptom severity estimation step includes detection and assessment of resting tremor (if no hand movement was detected) and assessment of bradykinesia (if hand movement was detected and gait was not detected). We removed periods of gait from bradykinesia analysis because motor symptoms associated with gait are generally assessed separately.^41^ Data from the wrist-worn device located on the most affected side spanning the duration (44.13 ± 10.53 min) between the start of first protocol activity and end of the last protocol activity during each visit (Supplementary Table 2) was used in this analysis. A detailed description of the experimental protocol and data analysis steps can be found in the Methods section.

### Training and performance of the context classifiers

#### Hand movement classifier

The video-based manual annotation process resulted in a total of 1186 3-s windows (118 goal directed (GD) and 1068 nongoal directed (NGD)) with hand movement and 807 3-s windows without hand movement. The hand movement classifier achieved an accuracy of 92.82% (weighted precision: 93%, recall: 93% and F1 score: 93%) with a false-negative rate of 4.72% and false-positive rate of 10.78%. The classification accuracy for GD movements and NGD movements was comparable (GD—sensitivity: 95.8%, false-negative rate: 4.2%; NGD—sensitivity: 95.2%, false-negative rate: 4.8%).

#### Gait classifier

We used a leave-one-subject-out cross validation approach with data from both healthy controls (HC) and PD subjects (*N* = 81) to assess performance of the gait classifier. Observations for the positive class (gait) were derived from two gait tasks (2.5-m walk and 10-m walk), whereas observations for the negative class (no gait) were derived from the remaining tasks with the exception of carry a book out and back 10 m (activities of daily living (ADL) 13) and carry a suitcase out and back 10 m (ADL 14; Supplementary Table 2). These ADL tasks involved short, sporadic periods of gait and were therefore excluded to ensure that the negative class only included non-gait observations. To address class imbalance, we randomly sampled the negative class to match the number of observations in the positive class. The final dataset included 9706 observations with a 50:50 split between the positive and negative classes. 24 (out of 47) features were retained after feature selection and used for training the gait classifier (Supplementary Table 3). The gait classifier achieved an accuracy of 96% (weighted precision: 96%, recall: 96% and F1 score: 96%) with a false-negative rate of 2.45% and false-positive rate of 6.4%. The difference in the classification accuracy between HC and PD subjects was not significant (95.96% vs. 95.32%). An ensemble of ten estimators (i.e., trees) was used for training the random forest model. We evaluated multiple settings for numbers of estimators (5, 10, 20, 50, and 100), but observed no significant improvement in model performance as we increased the number of estimators beyond 10.

### Training and performance of the resting tremor classifier

We implemented a gyroscope-based heuristic algorithm^15^ to generate labels for training the tremor classifier. To evaluate the reliability of the heuristic algorithm as a source of labels for tremor, we first assessed its performance on data from PD subjects. We found significant agreement between resting tremor constancy derived using the heuristic algorithm and clinical ratings of resting tremor constancy (MDS-UPDRS 3.18) provided by the live rater (Kruskal–Wallis chi-squared = 28.55, *p* < 0.0001). In addition, resting tremor constancy derived using this algorithm was able to significantly differentiate (*p* ≤ 0.05) between all pairs of clinical ratings except between 3 and 4 (*p* = 0.10). However, when we applied the algorithm to data from HC subjects, it resulted in a false positive rate of 10.21%.

**Fig. 2:**
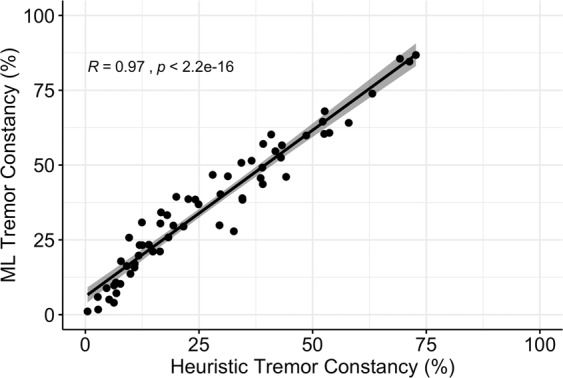
Agreement between the heuristic algorithm and ML classifier for resting tremor detection. Resting tremor constancy was estimated as a fraction of the total visit duration when resting tremor was detected from sensor data. Each point corresponds to one subject visit, solid line represents line of best fit and shaded region represents the confidence interval.

Consequently, we developed a binary resting tremor classifier using a machine learning (ML) approach and used data from HC subjects as the negative class (no tremor) and periods of tremor detected by the heuristic algorithm in PD subjects as the positive class for training. After feature selection, 18 features (out of 64) were retained for training the tremor classifier (Supplementary Table 3). Like the gait classifier, we evaluated multiple numbers of estimators (5, 10, 20, 50, and 100) but observed no significant improvement in model performance beyond 10. To evaluate the performance of the ML resting tremor classifier we used a leave-one-subject-out approach across all 81 subjects (50 HC and 31 PD). The classifier achieved an accuracy of 83% (weighted precision: 86%, recall: 86% and F1 score: 83%) across all subjects. As shown in Fig. 2, the predictions of the heuristic algorithm and ML classifier in PD subjects were strongly correlated (Pearson’s *R* = 0.97, *p* < 2.2e−16). In addition, there was a reduction in the false positive rate (6.83%) for the ML classifier in HC subjects.

### Agreement between clinical scores and sensor-derived measures

#### Resting tremor

**Fig. 3:**
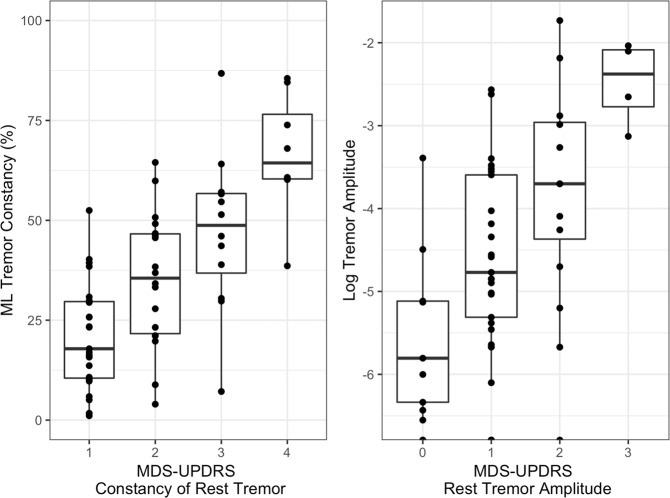
Sensor-based assessments of resting tremor and clinical ratings. Agreement between **a** sensor-derived measures of resting tremor constancy and MDS-UPDRS score of rest tremor constancy, and **b** sensor-derived measures of resting tremor amplitude and MDS-UPDRS score of rest tremor amplitude. Box plot center line, median; box limits, upper and lower quartiles; whiskers, 1.5× interquartile range; points, outliers.

Fig. 3a, b shows the agreement between sensor-derived measures and clinical ratings of resting tremor constancy (MDS-UPDRS 3.18) and amplitude (MDS-UPDRS 3.17). Because of a small number (<4) of samples, we grouped class 0 with class 1 for resting tremor constancy and class 3 with class 4 for resting tremor amplitude. We found good agreement between sensor-derived measures with clinical ratings of resting tremor constancy (Kruskal–Wallis chi-squared = 27.52, *p* < 0.0001) and resting tremor amplitude (Kruskal–Wallis chi-squared = 18.14, *p* = 0.0004). Post hoc Conover–Iman tests for pairwise comparisons with multiplicity adjustment using false-discovery rate correction revealed that the classifier was also able to significantly differentiate between adjacent pairs of clinical scores for resting tremor constancy and resting tremor amplitude (*p* ≤ 0.05), with the exception of scores 2 and 3 (*p* = 0.067) and 3 and 4 (*p* = 0.054) for resting tremor constancy (which were trending toward significance), and scores 0 and 1 (*p* = 0.096) and 2 and 3 (*p* = 0.1) for resting tremor amplitude.

#### Bradykinesia

Sensor-derived features of bradykinesia derived from data captured during unscripted activities (i.e., excluding MDS-UPDRS motor tasks) were compared with the hand bradykinesia clinical score (range: 0–12), which was derived by taking a sum of clinical scores of the most affected side for finger tapping (MDS-UPDRS 3.4), hand movements (MDS-UPDRS 3.5), and pronation-supination movements of the hand (MDS-UPDRS 3.6). The hand bradykinesia score was strongly negatively correlated (Pearson’s *R* = −0.69, *p* < 0.0001) with amplitude of hand movement measure and moderately correlated (Pearson’s *R* = 0.37, *p* = 0.003) with average length of bouts without hand movement measure.

**Fig. 4:**
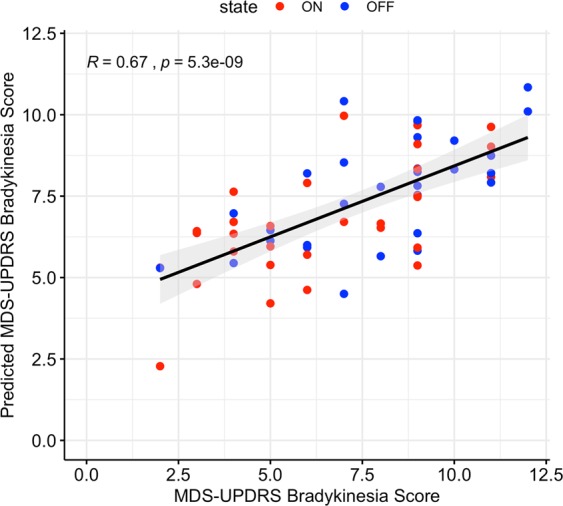
Sensor-based assessment of hand bradykinesia and clinical rating. Agreement between predicted hand bradykinesia score and live rater’s hand bradykinesia score. Each point corresponds to one subject visit, solid line represents line of best fit and shaded region represents the confidence interval. Hand bradykinesia score (range: 0–12) is a composite score derived by taking the sum of finger tapping (MDS-UPDRS 3.4), hand movement (MDS-UPDRS 3.5) and hand pronation-supination (MDS-UPDRS 3.6) scores.

We fit a longitudinal mixed effects model to predict the live rater’s hand bradykinesia score (see Methods for details). The optimal model included only one bradykinesia feature as predictor: amplitude of hand movements. This model predicted the live rater’s hand bradykinesia score with a RMS error of 2.01. As shown in Fig. 4, we observed a strong correlation (Pearson’s *R* = 0.67, *p* < 0.0001) between the predicted and live rater’s hand bradykinesia score.

### Treatment related changes in sensor-derived measures

**Fig. 5:**
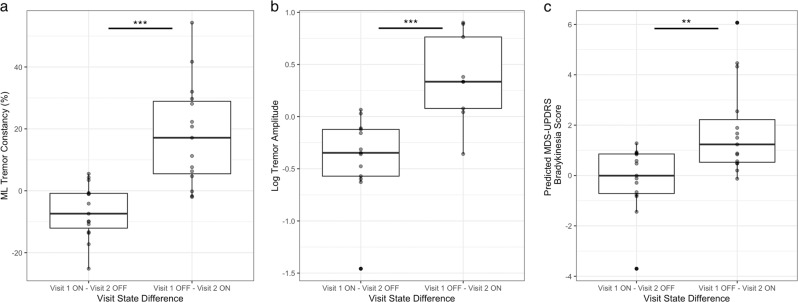
Motor state associated changes in sensor-based assessment of resting tremor and hand bradykinesia. Distribution of difference between ON and OFF states of sensor-derived measures of **a** tremor constancy, **b** tremor amplitude, and **c** predicted hand bradykinesia score grouped by the randomized order of motor state in visit 1. Box plot center line, median; box limits, upper and lower quartiles; whiskers, 1.5× interquartile range; points, outliers; **p < 0.01, ***p < 0.001.

To evaluate the effect of treatment related changes, we examined the relationship between patient reported motor state (ON—i.e., when motor symptoms are well controlled and OFF—i.e., when motor symptoms are poorly controlled) and sensor-derived measures of resting tremor and bradykinesia. Fig. 5a, b illustrates the distribution of differences between sensor-derived measures of resting tremor constancy and resting tremor amplitude between visit 1 and visit 2. We observed an increase in resting tremor constancy and amplitude for subjects who transitioned from an ON state to OFF state and vice versa for subjects who transitioned from an OFF state to ON state. A Wilcoxon signed rank test showed that changes in sensor-derived measures of resting tremor constancy and amplitude between the ON and OFF states were statistically significant (*p* < 0.0001 and *p* = 0.0002, respectively). The same trend was observed when analyzing the change in live rater score for resting tremor constancy and amplitude (*p* < 0.0001 for both tremor constancy and tremor amplitude).

Figure 5c illustrates the distribution of differences between the predicted hand bradykinesia score between visit 1 and visit 2. We observed a smaller median decrease in the predicted hand bradykinesia score for subjects who transitioned from an ON to OFF state compared to subjects who transitioned from an OFF to ON state. A Wilcoxon signed rank test showed that changes in predicted hand bradykinesia score between the ON and OFF states were statistically significant (*p* = 0.009). When comparing each of the four sensor-derived features with the ON and OFF states, only amplitude of hand movement feature showed statistically significant changes between each state (Wilcoxon *p* = 0.008). We observed an increase in live rater’s hand bradykinesia score for subjects who transitioned from an ON state to OFF state and vice versa for subjects who transitioned from an OFF state to ON state (*p* = 0.0005).

For both tremor and bradykinesia, we observed that the magnitude and variability of the change in sensor-derived measures as subjects transitioned from the OFF to ON state was larger than when subjects transitioned from the ON to OFF state. This can be explained by the fact that the time since last levodopa dose in the OFF state for subjects who transitioned from an OFF to ON state (889.87 ± 160.66 min) was significantly longer (*p* = 0.0002) compared to subjects who transitioned from ON to OFF state (mean: 497.89 ± 270.04 min). Time since levodopa was not significantly different (*p* = 0.42) in the ON state for the two groups (ON to OFF and OFF to ON).

### Agreement between live rater and video raters

Video-based assessments performed using telemedicine can be an alternative approach to capturing more data outside of the clinic. However, while there is evidence of satisfactory inter-rater reliability of the motor examination section of the UPDRS between live raters,^42^ the agreement between live and video-based motor examination is not well understood. To investigate this, we examined the agreement between ratings provided by the live rater (during clinic visits) and by video raters who were blinded to when the patients took their medications. As shown in Table 1, for tremor constancy and tremor amplitude, the level of agreement between the live and video ratings as measured by Cohen’s Kappa *K* was poor (*K* < 0.39) to fair (*K* < 0.59) whereas agreement between the two video raters was moderate (*K* > 0.6). For hand bradykinesia score, we observed a moderate to good agreement as measured by the intraclass correlation coefficient (ICC) (0.5 < ICC < 0.75) between the live and both video raters as well as between the two video raters. These results point to the challenges of using a subjective rating scale like the MDS-UPDRS for performing video-based remote assessments.

**Table 1:** Comparison of video raters and live rater for tremor and hand bradykinesia MDS-UPDRS scores.

Comparison	Score	Statistic
Live Rater vs. Video Rater 1	Tremor Constancy	*K* = 0.325, *p* < 0.0001
	Tremor Amplitude	*K* = 0.395, *p* < 0.0001
	Hand Bradykinesia	ICC = 0.704, (0.377, 0.848)
Live Rater vs. Video Rater 2	Tremor Constancy	*K* = 0.317, *p* < 0.0001
	Tremor Amplitude	*K* = 0.45, *p* < 0.0001
	Hand Bradykinesia	ICC = 0.565, (0.101, 0.784)
Video Rater 1 vs. Video Rater 2	Tremor Constancy	*K* = 0.620, *p* < 0.0001
	Tremor Amplitude	*K* = 0.623, *p* < 0.0001
	Hand Bradykinesia	ICC = 0.61, (0.419, 0.75)

### Patient acceptance of wearable devices

**Fig. 6:**
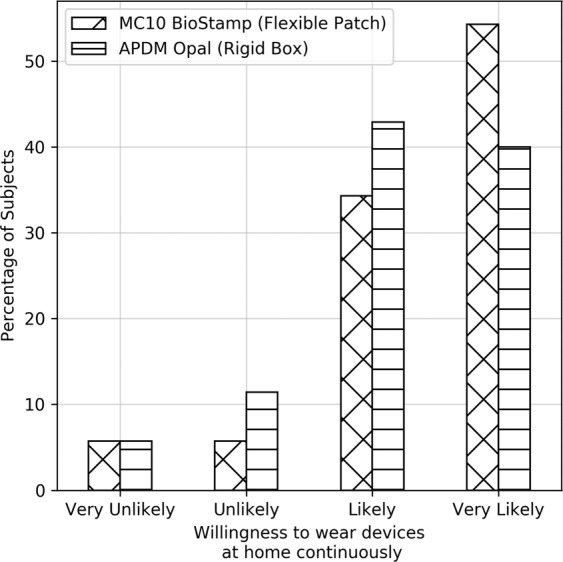
Willingness of subjects to wear rigid (Opal) or flexible patch-like (BioStamp) devices continuously at home. A marked preference for a flexible patch-like device was observed for subjects who were very likely to wear devices continuously at home.

Patient acceptance is essential for the viability of wearable devices as tools for continuous monitoring of motor symptoms. In this study, we used devices that came in two form factors: rigid box (APDM Opal) and flexible patch (MC10 BioStamp). Although the analysis presented in this work is based on data from a single wrist-worn sensor (APDM Opal), we assessed wearability of all devices worn by patients (11 devices at multiple body locations). To assess wearability, a questionnaire was administered after subjects completed the second in-clinic visit. Subjects were asked to rate their willingness to wear the devices continuously for an extended period of time, overall comfort of the devices, and whether any specific sensor locations were uncomfortable. Approximately, 85% of subjects were either likely or very likely to wear the sensors for an extended period of time (Fig. 6). Of the subjects that were very likely to wear the devices for an extended period of time and reported them very comfortable, there was a marked preference (54.3% vs. 40%) for the flexible patch form factor. While both type of devices were rated highly by subjects for comfort, 7 out of 8 subjects reported sternum as the most uncomfortable location for devices with a rigid form factor; whereas 3 out of 4 subjects reported flexible patches placed on the lower extremity (thigh and ankle) as uncomfortable. We observed a high level of acceptance for the wrist location for either device types.

## Discussion

**Fig. 7:**
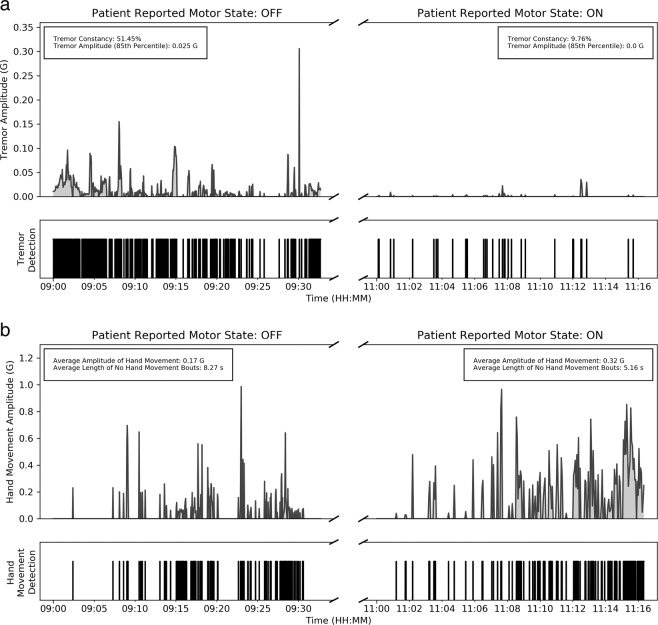
An example showing continuous assessment of resting tremor and bradykinesia using accelerometer data from a device worn on the most affected wrist. Data was collected on one PD patient across two in-lab visits. The first visit was in the OFF state and second visit in the ON state. **a** Continuous tremor detection and tremor amplitude measurement in the OFF (left panel) and ON (right panel) state. Inset shows aggregate measures of tremor constancy and tremor amplitude. Tremor constancy was rated 3 in the OFF state and 1 in the ON state. Tremor amplitude was rated 2 in the OFF and 0 in the ON. **b** Continuous hand movement detection and hand amplitude measurement in the OFF (left panel) and ON (right panel) state. Inset shows aggregate measures of hand movement amplitude and length of no hand movement bouts. Hand bradykinesia was rated 9 in the OFF state and 2 in the ON state.

We investigated the use of accelerometer data captured by a single wrist-worn device for monitoring resting tremor and bradykinesia in patients with PD. The proposed method was able to derive clinically meaningful measures of symptom severity from data collected during the performance of unscripted activities during in-clinic visits. An example of the continuous measures produced by the proposed method is shown in Fig. 7. As the subject transitions from the OFF to ON state, we can observe a marked decrease in the constancy and amplitude of resting tremor, and a marked increase in the amount and amplitude of hand movement. The motivation behind using a hierarchical framework to identify context and then assess motor symptoms was to provide more interpretable data during unsupervised ambulatory monitoring. However, because of the hierarchical relationship between context detection and symptom assessment, there is a risk of error propagation if mistakes are made early on. While the false positive rates for hand movement (10.78%) and gait (6.4%) classifiers did not appear to have a significant impact on motor symptom assessment, application of this method to data collected over longer durations (days or weeks) is necessary to investigate its generalizability.

By assessing agreement of sensor-derived measures with clinical assessments (MDS-UPDRS) and PD motor states (ON–OFF), we were able to assess criterion and discriminative validity of the proposed method. While maintaining high sensitivity, a random forest classifier trained for classifying resting tremor using features extracted from accelerometer data was able to achieve a 3.4% reduction in false-positive rate compared to a previously published gyroscope-based heuristic algorithm.^15^ This translates into sensor-derived tremor constancy of 4.42 ± 2.64% (mean ± std.) in the HC subjects, which is close to the level that would be considered a score of 0 on the UPDRS III rating of tremor constancy. The improvement in specificity can be largely attributed to (1) the use of ML to learn the rules in a higher dimensional feature space, and (2) the inclusion of data from HC subjects as the negative class during training. While completely eliminating false positives might not be feasible, future efforts should focus on further refinements that could help reduce the false positive rate. One approach could be to apply post-processing rules for removing isolated tremor windows at the cost of reducing sensitivity. Alternatively, the classification threshold could be tuned to increase specificity (i.e., lower false-positive rate) at the cost of reducing sensitivity. The suitability of an approach will depend on the application scenario and a careful analysis of tradeoffs. Sensor-derived measures for both resting tremor constancy and amplitude showed good agreement with respective clinical measures and demonstrated excellent ability to discriminate between ON–OFF motor states. However, we observed a tendency of sensor-derived estimates to underestimate at higher scores. For example, the sensor-derived estimate of tremor constancy was between 75–100% for only two observations out of 7 that had been assigned a clinical score of 4. One potential reason for this could be that while sensor-derived measures are based on the entire observation period (i.e., entire visit), clinical scores might be influenced by what the live rater observes in a period closer to the point of assessment.

Of the four sensor-derived bradykinesia measures that were extracted during periods of voluntary hand movement, most of the variance in the hand bradykinesia score was explained by the hand movement amplitude feature (48%) followed by the no hand movement bouts feature (14%). A possible explanation for this is that when raters are scoring bradykinesia items of the MDS-UPDRS, movement amplitude is the primary factor in their assessment whereas aspects such as smoothness of hand movements or amount of hand movement observed during the assessment period are discounted. Using a longitudinal mixed-effects model we were able to achieve RMSE of 2.01 for predicting the hand bradykinesia score (range: 0–12) and 7.57 for predicting the total bradykinesia score (range: 0–44). The increased RMSE for the total bradykinesia score can be explained by the fact that a single wrist worn device is limited to the most affected side and not able to measure bradykinesia on the contralateral arm or lower limbs. Future efforts should be aimed at investigating the tradeoff between improving sensitivity of the sensor-derived measures and increasing system complexity by using multiple devices.

A high degree of compliance is essential for deployment of wearable device based digital endpoints. An advantage of the proposed method is that it relies on a single, power efficient accelerometer sensor, which can be deployed in a wristwatch-like device that does not require frequent charging. However, while we observed a strong preference for wrist-worn devices, in a large study (*N* = 953) aimed at assessing feasibility of using wearable devices for long-term (monitoring duration ranged from 6 to 13 weeks) continuous monitoring of PD motor symptoms, the median compliance for the wrist-worn device was only 65%.^43^ Therefore, considerations for human factors like esthetics, comfort, and usability in the design of wearable devices will play a critical role in ensuring compliance, and ultimately determine the quality and quantity of data that can be collected.

Clinical trials aimed at improving the management of PD motor symptoms largely rely on the use of subjective endpoints to assess the efficacy of new therapies. These endpoints, which are typically based on either infrequent clinical assessments or unreliable patient self-reports, can fail to capture the impact of disease on daily life.^44^ Wearable devices, which can provide objective and high resolution monitoring of motor symptoms, have the potential to deliver new insights that can transform the development of new therapies as well as improve clinical management of PD.^39^ However, deploying wearable devices under free-living conditions comes with a unique set of challenges. First and foremost, will the performance of methods developed based on data collected in constrained or semi-constrained settings (e.g., clinic or research lab) be generalizable to free-living conditions? This question of ecological validity is further compounded by factors such as compliance with the use of wearable devices, as well as errors in device setup (e.g., device placed at the wrong body location), which could impact the data quantity and quality. Furthermore, the heterogeneity of PD^45^ implies that wearable devices will only be able to provide a partial picture of the clinical features that are experienced by a patient. Therefore, it will be important to develop digital tools such as smartphone apps that can capture a more holistic representation of various motor^19^ and nonmotor^46^ symptoms. Subjective clinical assessments (e.g., MDS-UPDRS) and patient self-reports (e.g., motor diaries) are typically used to validate digital assessment tools. While it is necessary to demonstrate agreement between a new digital tool (objective and continuous) and currently accepted methods (subjective and episodic), there is a fundamental difference between the type of information that is captured by the two approaches. An opportunity for the research community is to come up with new objective measures or scales along with target ranges that can be used for clinical decision-making.^47^

While there are practical advantages to minimizing the number of devices, because PD motor symptoms can manifest across the body, application of the proposed approach may be limited to monitoring patients who have not developed axial symptoms like gait impairments and postural instability. Consequently, multiple devices and sensing modalities (e.g., gyroscopes and electromyography) might be essential for quantifying motor symptoms in patients with advanced PD. Data were collected in an in-clinic setting from a small sample of PD and HC subjects who were not age or gender matched. The impact of this mismatch was partly mitigated by the fact that (a) data from HC subjects only were used for training the tremor classifier as the negative class (no tremor) and, (b) a closer examination of the performance of the gait classifier revealed comparable accuracy for the PD (95.32%) and HC (95.96%) subjects. Nonetheless, further validation of this approach in a semi-constrained or unconstrained setting in a larger population is needed before the system can be deployed at home. Experimental conditions might also have had an influence on motor behavior of subjects (for both HC and PD). Besides being in the clinic under observation of the study staff, subjects had to deal with the physical and psychological aspects associated with wearing 11 devices at multiple locations on the body. While it has previously been shown that stress can have an impact on tremor characteristics^48^ and there is some evidence that motivation can be helpful in overcoming bradykinesia,^49^ it is not possible to directly assess the impact of the experimental conditions on motor symptoms in this study.

PD motor symptoms fluctuate on a continuum between the ON and OFF state. Data were only collected in the ON and OFF state, which limited our ability to assess criterion validity during the transition period. Future studies could be designed to capture wearable sensor data continuously under free-living conditions along with periodic clinical assessments (e.g., every 30 min) that provide anchor points for assessing criterion validity. Finally, minimizing motor fluctuations and dyskinesias^50^ is a significant focus area for disease management and development of new therapies because of their impact on quality of life and functional abilities of patients. Because only a few subjects in our study experienced dyskinesias, we were not able to develop an approach to detect and quantify dyskinetic movement.

## Methods

### Study design

Data from HC and PD patients was collected in two studies with similar experimental protocols.^51^ Both studies consisted of two in-clinic visits each lasting approximately one hour. Visits were separated by a few hours on the same day or by up to 14 days depending on subject preference. For HC both visits were identical whereas PD patients were randomly assigned to be in their self-reported ON (i.e., when motor symptoms are well controlled) or OFF (i.e., when motor symptoms are poorly controlled) motor state^52^ for a given visit. For PD subjects who completed both visits on the same day, if visit 1 was in the ON state, visit 2 (OFF state) began 0.5–1 h before the next scheduled l-Dopa dose, whereas if visit 1 was in the OFF state, visit 2 (ON state) began with the subject taking their l-Dopa dose with ON/OFF questioning every 0.5 h until ON state was confirmed or 1.5 h post-dose. The washout period for PD subjects who were OFF in visit 1 was 889.87 ± 160.66 min compared to 497.89 ± 270.04 min for subjects who were OFF in visit 2. The study had approval from the Tufts Medical Center and Tufts University Health Sciences Institutional Review Board. All participants in the study gave written informed consent prior to enrollment.

### Instrumentation

**Fig. 8:**
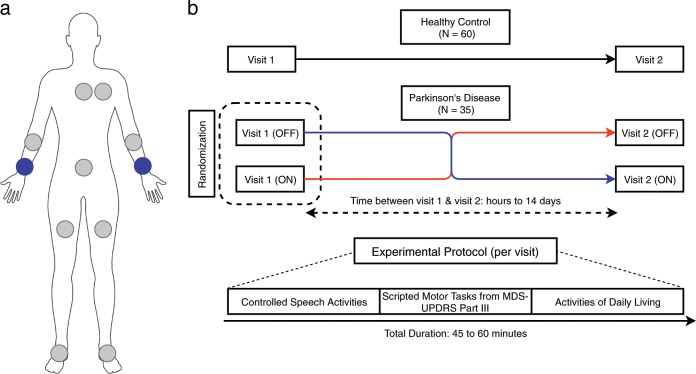
Sensor setup and experimental protocol. **a** An illustration of the sensor setup used for data collection. Sensor data from only one device (blue) attached to the wrist of the most affected side (PD) or dominant side (HC) was used for the analysis presented in this paper. **b** Both the PD study and the HC study consisted of two in-clinic visits. Subjects performed a series of activities that were repeated in each visit. Activities were categorized into controlled speech activities, scripted MDS-UPDRS tasks, and activities of daily living. For HC’s both visits were identical whereas PD patients were randomly assigned to be in their self-reported ON or OFF motor state for a given visit.

Subjects wore multiple devices with inertial sensors (three-axis accelerometer, thre-axis gyroscope, and three-axis magnetometer) on their arms, legs, and torso as illustrated in Fig. 8a. Devices included Opal (APDM, Inc.), and BioStamp (MC10, Inc.) devices. The analysis presented in this work is based on inertial sensor data recorded by Opal devices located on the wrist (dominant side for HC’s and most affected side for PD patients). Sensor data included triaxial accelerometer, gyroscope, and magnetometer recordings sampled at 128 Hz. In addition, video recordings of all study activities were performed for later review.

### Subjects

A total of 60 HC subjects (Age: 44.1 ± 10.70 [23–69] years; Sex: 27 M/33 F) and 35 PD subjects with mild to moderate PD (Age: 68.31 ± 8.03 [46–79] years; Sex: 23 M/12 F; Hoehn & Yahr stage < = 3 (H & Y I/II/III: 2/26/7); MDS-UPDRS III: 52.86 ± 16.03) participated in the two studies. All PD patients were taking Levodopa and could recognize their “wearing off” symptoms. The HC population had no known sensory or motor deficits. For the analysis presented herein, data from a total of 50 HC (Age: 43.9 ± 10.02 (23–64), Sex: 23 M/27 F) and 31 PD subjects (Age: 68.1 ± 8.13 (46–79), Sex: 20 M/11 F) were used. Detailed subject characteristics are listed in Table 2. Four PD and ten HC subjects were excluded from the analysis because of issues with time alignment of sensor data with protocol activities or missing data from the wrist sensors. Time alignment issues resulted from a mismatch between clocks associated with wearable devices and video recordings, which prevented ground truth labels being generated for various activities performed during the experimental protocol.

**Table 2:** Characteristics of subjects used for analysis.

Characteristic	HC (*n* = 50)	PD (*n* = 31)
M/F (*n*)	23/27	20/11
Age (years)	43.9 ± 10.02	68.1 ± 8.13
Levodopa Equivalent Daily Dose (mg/day)	–	157.81 ± 80.92
Time Since First Diagnosis (years)		5.62 ± 3.46
Hoehn and Yahr (n)	–	H & Y I–2 H & Y II−24 H & Y III−5
MDS-UPDRS III	–	50.55 ± 15.53
Tremor Constancy (MDS-UPDRS 3.18)	–	2.16 ± 1.25
Tremor Amplitude (MDS-UPDRS 3.17)	–	1.42 ± 0.91
Finger Tapping (MDS-UPDRS 3.4)	–	2.65 ± 0.86
Hand Movements (MDS-UPDRS 3.5)	–	2.42 ± 0.98
Pronation Supination (MDS-UPDRS 3.6)	–	2.97 ± 0.86
Hand Bradykinesia (MDS-UPDRS 3.4 + MDS-UPDRS 3.5 + MDS-UPDRS 3.6)	–	8.03 ± 2.4

### Experimental protocol

The experimental protocol comprised of two visits to the study site (Fig. 8b). During each visit, subjects were instructed to perform a series of tasks (Supplementary Table 2). These tasks can be broadly divided into activities of daily living (ADL), controlled speech activities (CSA), and tasks from the motor assessment section of the MDS-UPDRS Part III.^9^ ADL tasks were selected because they involved a combination of gross mobility (e.g., carrying a book) and fine coordination (e.g., writing a sentence),^53^ which are typically performed during daily life. The duration of these tasks ranged from ~5 s to ~3 min, with each visit totaling ~45–60 min. A neurologist administered Part III of the MDS-UPDRS and rated the items on a scale from 0 to 4^9^ (live rater). In addition, video recordings of these assessments were retrospectively reviewed and scored by two neurologists (video raters). Video raters were blinded to the treatment state (ON or OFF) of PD subjects.

### Detection and assessment of motor behavior

We chose a 3-s window length for the gait and tremor classifier based on prior work^14,54^ in the area of monitoring PD symptoms and human activity recognition that has shown that this duration provides sufficient resolution for extracting relevant time and frequency domain features.^55^ Because tremor has a fundamental frequency typically between 3–8 Hz, a 3-s window length should be sufficient^14^ for capturing signal features associated with high frequency movements. For the gait classifier, considering normative values of cadence (>102 ± 11 steps/min) in older adults,^56^ we can expect that a 3-s window would be able to capture multiple steps. Furthermore, a systematic analysis^54^ of effect of window length on accuracy of human activity classification revealed that a minimum window length of 1–2 s is required to achieve a good tradeoff between classification accuracy and inference speed.

### Hand movement classifier

To assess performance of the hand movement classifier, we randomly selected ten PD subjects and manually annotated (marked the start and end) periods involving hand movement by reviewing video recordings. This choice was motivated by the common ML practice of using 20–30% of data for testing. Annotations were performed by three raters and reviewed by an arbitrator for accuracy. Data from the following tasks were annotated: conversation (CSA 1), kinetic tremor of the hands (MDS-UPDRS 3.16), pronation/supination movements of the hand (MDS-UPDRS 3.6), bottle shake (ADL 7) and gait (MDS-UPDRS 3.10; Supplementary Table 2). These tasks were selected because they provided a good representation of different types of hand movements that are performed in daily life. Hand movements were annotated as either GD (e.g., reaching for a glass of water) or NGD (e.g., hand gestures while having a conversation or during gait)^57^ movements. The distinction between GD and NGD was made to assess if the type of hand movement had an impact on the performance of the classifier.

**Fig. 9:**
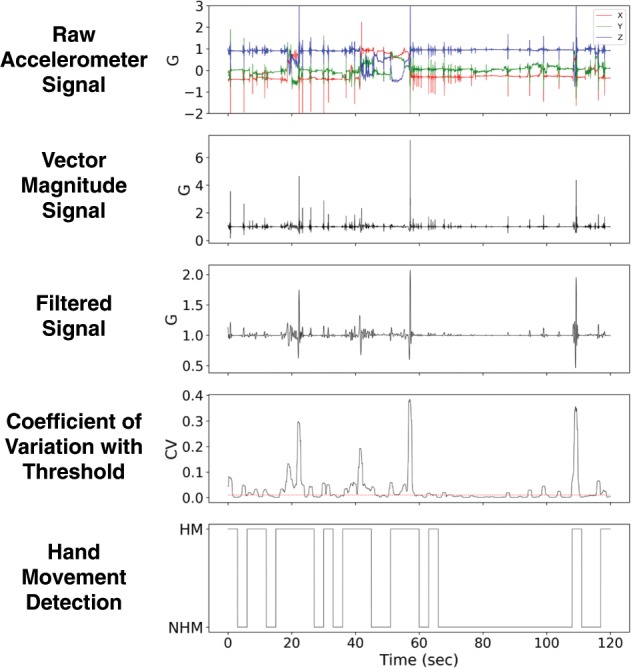
Method for detecting hand movement from raw accelerometer data. Vector magnitude derived from raw triaxial accelerometer data is filtered to remove high frequency components. An empirically derived threshold is then applied to coefficient of variation of the filtered signal to detect hand movement. HM, hand movement; NHM, no hand movement.

Sensor data acquired during the performance of these tasks were then segmented into 3-s nonoverlapping windows. A window was labeled as hand movement if more than 50% of its duration was marked as either GD or NGD movement. For each 3-s window, raw accelerometer data was first processed by first taking the vector magnitude ($\sqrt {x^2 + y^2 + z^2}$) to remove dependence on device orientation. This was followed by low-pass filtering the resulting signal using a sixth order Butterworth infinite impulse response (IIR) filter with a 3 Hz cutoff frequency to attenuate high frequency movements typically associated with tremor. We then computed a rolling coefficient of variation ($c_v = \sigma /\mu ,{\rm{where}}\;\sigma = {\rm{standard}}\;{\rm{deviation}},\mu = {\rm{mean}}$) by sliding a 1-s window across the filtered signal to create an envelope like representation of the signal. A threshold of 0.01 was determined empirically and any coefficients of variation values above this threshold were marked as periods of hand movement. The resulting binary time series (0 = no hand movement, 1 = hand movement) was then divided into nonoverlapping 3-s windows. If more than 50% of the samples in a window were marked as hand movement, then that window was labeled as a hand movement window. Figure 9 shows an example of the output at each processing step of the hand movement detection method.

#### Gait classifier

We trained a binary machine learning (ML) classifier to detect periods of gait from the raw accelerometer data. Observations of the positive class (gait) were derived from two gait tasks (2.5-m walk and 10-m walk) whereas the remain tasks (excluding the ADL tasks that included walking) from each visit were used to derive observations of the negative class (not gait). All data from the available HC and PD subjects were used for training the gait classifier model.

The pipeline for training the gait classifier, included steps for preprocessing, feature extraction, feature selection, and model training/evaluation. The raw acceleration data was band-pass filtered using a first order Butterworth IIR filter with cutoff frequencies of 0.25–3.0 Hz to attenuate high frequency movements associated with tremor. We then projected the band-pass filtered three-axis accelerometer signals along the first principal component derived using principal component analysis (PCA) to generate a processed signal that is independent of device orientation. These preprocessing steps yielded 4 processed time series of acceleration signals (3 band-pass filtered signals and 1 PCA projection). The signals were then segmented into 3-s nonoverlapping windows and a total of 47 time and frequency domain features (listed in Supplementary Table 3) were extracted from each window. The number of observations was then randomly sampled to balance both the positive and negative classes prior to the feature selection step. Feature selection was performed using recursive feature elimination with cross-validated selection of the optimal features using a decision tree classifier.^58^ We then trained a random forest classifier using the selected features. A leave one subject out approach was used to assess the performance (accuracy, precision, recall, and F1 score) of the gait detection model.

#### Resting tremor

Assessment of resting tremor is based on two measures: constancy and amplitude. Constancy measures the percentage of time that tremor is present whereas amplitude measures how fast and big the tremor related movements are. We trained a binary machine learning classifier to detect periods of tremor from raw accelerometer data. To generate labels for training the classifier, we implemented a heuristic algorithm^15^ based on angular velocity signals measured by the three-axis gyroscope (on the same device) as an alternative to generating labels by manually annotating the video recordings. The heuristic algorithm is straightforward to implement and has been shown^15^ to be very accurate (99.5% sensitivity and 94.2% specificity) when compared to video reference. While the heuristic algorithm might not provide perfect labels, it is preferable to manually annotating videos, which is extremely time consuming, error prone and suffers from inter and intra rater reliability issues.^59^ To generate labels for the positive class (tremor present), we applied the heuristic algorithm to continuous stream of sensor data from the entire visit for PD subjects. We then segmented the data into 3-s nonoverlapping windows. Any 3-second window where tremor was present for more than 50% of the duration was assigned a positive label. To generate labels for the negative class (no tremor), we used all available data from HC subjects. This was justified by the fact that HC subjects were significantly younger than PD subjects (*p* < 0.0001) and had no sensory or motor impairments.

The pipeline for training a tremor classifier based on three-axis accelerometer data, included steps for preprocessing, feature extraction, feature selection, and model training and evaluation. The first step in the pipeline generated multiple processed signals by applying filtering and dimensionality reduction to the raw acceleration signals. Processed signals were first derived by applying a first order Butterworth IIR band-pass filter in the nontremor movement (cutoff: 0.25–3 Hz) and tremor (cutoff: 3.5–7.5 Hz) band. The first principal component was calculated from the filtered signals in both the nontremor movement and tremor movement bands. The first principal component was included as a processed signal for feature extraction to reduce dependence on device orientation. These preprocessing steps resulted in eight processed signals (i.e., three signals in the tremor movement band, three signals in the nontremor movement band, one PCA projection in the tremor movement band, and one PCA projection in the non-tremor movement band).

The 8 processed signals were then segmented into 3-s nonoverlapping windows and a total of 64 time and frequency domain features (listed in Supplementary Table 3) were extracted from each window. Observations were then randomly sampled to balance both the positive and negative classes prior to feature selection. Feature selection was performed using recursive feature elimination with cross-validation using a decision tree estimator.^58^ We then trained a random forest classifier using the selected features. A leave one subject out approach was used to assess the performance (accuracy, precision, recall, and F1 score) of the tremor detection model.

Tremor amplitude was calculated for each 3-s window that was classified as tremor. First, in order to attenuate acceleration related to movements outside the tremor band, the raw accelerometer data was band-pass filtered in the tremor band with a third order Butterworth IIR filter with cutoff frequencies of 3.5–7.5 Hz. The vector magnitude ($\sqrt {x^2 + y^2 + z^2}$) of the filtered signal was then computed to remove dependence on device orientation as well as directionality of tremor movements. Tremor amplitude (unit: g) was then calculated by computing the root mean square (RMS) of the vector magnitude signal for each 3-s window. To estimate the tremor amplitude value for the entire visit, we calculated the 85th percentile value across all 3-s windows within the duration of the visit. The percentile value was determined by assessing the correlation with the tremor amplitude score in increments of 5% from 80th percentile to 95th percentile. This was done to mimic the clinical assessment of tremor amplitude, which is based on the maximum amplitude observed by the examiner during the assessment period.

#### Bradykinesia

The assessment of bradykinesia is based the examiner’s observation of slowness, hesitancy and amplitude of movement as well as poverty or absence of movement.^9^ To capture these aspects of bradykinesia, we derived four measures from raw accelerometer data: amplitude of hand movements (amplitude and slowness), smoothness of hand movements (hesitancy), percentage of time spent with no hand movement (poverty or absence), and average length of bouts with no hand movement (poverty or absence). These four measures were calculated when hand movement was detected from data collected during the entire visit except during scripted MDS-UPDRS Part III tasks. MDS-UPDRS Part III tasks were excluded because they required subjects to engage in highly prescribed motor tasks, which were often performed at the subject’s maximum capacity (e.g., a typical MDS-UPDRS Part III instruction would be “perform pronation-supination of the hand as fast and wide as you can”). A given 3-s window was used for calculating bradykinesia measures if hand movement was present (hand movement classifier = YES) and the subject was not walking (gait classifier = NO).

Aspects associated with poverty or absence of hand movement were captured by measuring percentage of time spent without hand movement as well as average duration of periods without hand movement. Longer average duration of periods without hand movement would indicate that long stretches of time were spent without movement whereas shorter duration would indicate that movements are frequent. Percentage of time spent with no hand movement was calculated by dividing the aggregate duration of 3-second windows with no hand movement (hand movement classifier = NO) by the total duration of data used for analysis. Average length of bouts with no hand movement was calculated by taking the mean value of contiguous blocks of windows that were classified as no hand movement (hand movement classifier = NO).

Amplitude of hand movement was derived as a measure of both amplitude and slowness as accelerometer measurements at the wrist would respond to how small and slow the hand movements are. For example, large but slow movements would generate lower levels of acceleration compared to small but fast movements. Smoothness of hand movement captures aspects associated with hesitancy during performance of a task. Acceleration signals generated during performance of fluid motions will be smoother compared to those with hesitations and halts. To calculate amplitude and smoothness measures, raw accelerometer data was first band-pass filtered using a fourth order Butterworth IIR filter with cutoff frequencies of 0.25–3.5 Hz to attenuate tremor related movements. For each 3-s window we then calculated the RMS value of the vector magnitude ($\sqrt {x^2 + y^2 + z^2}$) of the filtered accelerometer signal. Amplitude of hand movement was calculated by taking the mean of the RMS values across all 3-s windows within the duration of the visit. Smoothness of hand movements was calculated by computing the mean squared jerk^60^ (i.e., rate of change of acceleration), which was scaled by the maximum value of the vector magnitude signal and duration for each 3-s window. The value of mean squared jerk will be higher for movements that involve sudden and frequent changes in direction of movement and lower for movements that are smooth and fluid. To estimate jerk measure for the entire visit, we calculated the 95th percentile value of mean squared jerk across all 3-s windows within the duration of the visit. The percentile value was determined by assessing the correlation with the hand bradykinesia score in increments of 5% from 80th percentile to 95th percentile.

### Statistical methods to measure agreement between sensor-derived measures and clinical assessments

Shapiro–Wilk test was used to assess normality of features. Since features were not normally distributed, nonparametric statistical methods were used throughout. Variation of features with the live rater’s item score was quantified by the Kruskal–Wallis test. Post hoc Conover-Iman tests were used for pairwise comparisons and multiplicity was adjusted using false-discovery rate correction. Pairwise differences in tremor and bradykinesia features between ON and OFF states, and between transitions from ON to OFF and OFF to ON were tested using Wilcoxon rank sum test.

Bradykinesia features were used to fit a longitudinal mixed effects regression model to predict the live rater’s bradykinesia score using the four sensor-derived bradykinesia features as well as visit number, subject specific covariates, namely gender and years since first symptoms as fixed effects, and subject as random effect. Stepwise model selection was performed using Akaike Information Criterion as a cost function to achieve the optimal model fit. The accuracy of the model prediction based on its fixed effects was tested via leave-one-subject-out cross validation.

Agreement between the video raters and live rater for tremor constancy and amplitude (which are ordinal ratings from 0 to 4) was assessed using Cohen’s Kappa *K* with linear weights.^61^ The hand bradykinesia score was considered as a continuous variable ranging from 0 to 12, and hence the ICC was computed as a measure of agreement between raters. ICC estimates and their 95% confidence intervals were calculated using the R package *irr* based on a 2-way mixed-effects model using “agreement” as the ICC type.^62^

### Reporting summary

Further information on research design is available in the Nature Research Reporting Summary linked to this article.

## Supplementary information

Supplementary Material

Reporting Summary

## Data Availability

The datasets used in this study are not publicly available because they contain protected patient health information. Derived feature table with tremor and bradykinesia measures extracted for each PD subject per visit can be made available from the corresponding author upon reasonable request.

## Code availability

The code used to create the classifiers outlined in this publication is available on GitHub (https://github.com/NikhilMahadevan/analyze-tremor-bradykinesia-PD).
