# Supplementary material for: Development of digital biomarkers for resting tremor and bradykinesia using a wrist-worn wearable device
Source: NPJ Digit Med. 2020 Jan 15;3:5. doi: 10.1038/s41746-019-0217-7 (PMC6962225; doi:10.1038/s41746-019-0217-7)
Supplement: Supplementary file 1 — Supplementary Material [file 41746_2019_217_MOESM1_ESM.pdf]

Supplementary Information

Supplementary Table 1. Related work.

Work	Year	# Patients		Symptoms		Sensors	# Devices	Locations	Data Collection	Criterion Validity	Discriminative Validity	Utility	Algorithm
		PD	Controls	Tremor	Brady-kinesia								
Hoff et al.	2004	15		Yes	Yes	Accelerometer	3	Sternum, wrist and thigh on most affected side	24 hour recording Motor diaries	No	Yes	No	Heuristic algorithm based on empirically derived thresholds.
Keijsers et al.	2006	23		Yes	Yes	Accelerometer	6	Both upper arms and upper legs, sternum, wrist on most affected side	35 ADLs ~3hr monitoring period (ON/OFF)	No	Yes	No	Heuristic algorithm based on empirically derived thresholds.
Salarian et al.	2007	21	10	Yes	Yes	Gyroscope	2	Both wrists	15 ADLs + alternating hand movement (right and left)	Yes	Yes	No	Heuristic algorithm based on empirically derived thresholds.
Zwartjes et al.	2010	6	7	Yes	Yes	Accelerometer Gyroscope	4	Sternum, wrist, thigh and foot on the most affected side	8 ADLs + 5 motor assessment tasks	Yes	Yes	No	Heuristic algorithm based on empirically derived thresholds.
Griffiths et al.	2012	34	10	No	Yes	Accelerometer	1	Wrist on the most affected side	ADLs and constrained motor tasks in lab Upto 10 days at home	Yes	No	No	Proprietary. Heuristic algorithm based on empirically derived thresholds.

Rigas et al.	2012	18	5	Yes	No	Accelerometer	6	Both wrists and ankles, sternum, waist	5 ADLs + 3 motor assessment tasks	Yes	No	No	Posture recognition and tremor severity estimation algorithm trained using machine learning (HMM).
Roy et al.	2013	19	4	Yes	No	Accelerometer EMG	4	Both forearms and shanks	Self selected ADLs	Yes	No	No	Symptom detection algorithm trained using machine learning (DNN). MAP classifier for estimating severity of symptoms.
Tzallas et al.	2014	24(in lab) 12 (at home)	5	Yes	Yes	Accelerometer Gyroscope	5	Both wrists and ankles, waist	Prescribed and self-selected ADLs In lab: ~15 minutes/subject At home: ~8 hours/subject	Yes	No	Yes	Machine learning classifiers for assessment of tremor, bradykinesia and dyskinesia
Horne et al.	2015	64	38	No	Yes	Accelerometer	1	Wrist on the most affected side	Upto 10 days at home	Yes	Yes	No	Proprietary. Heuristic algorithm based on empirically derived thresholds (Griffiths et al. 2012).

Braybrook et al.	2016	194	28	Yes	No	Accelerometer	1	Wrist on the most affected side		No	Yes	No	Heuristic algorithm based on empirically derived thresholds.
Pulliam et al.	2018	13		Yes	Yes	Accelerometer Gyroscope	2	Wrist and ankle on the most affected side	6 ADLs (hygiene, eating, dressing, desk work, entertainment, laundry) ON/OFF	No	Yes	No	Proprietary. Heuristic algorithm based on empirically derived thresholds.
Rodríguez-Molinero et al.	2018	23		No	Yes	Accelerometer	1	Waist	1 - 3 days at home Motor diaries	No	Yes	No	Machine learning classifier for gait detection. Heuristic algorithm for bradykinesia assessment based on movement fluidity during gait using patient specific threshold.

Supplementary Table 2. List of activities performed in both PD and HC studies.

Scripted MDS-UPDRS-III Activities	Activities of Daily Living	Controlled Speech Activities
(3.3) Rigidity (neck, wrist/elbow and hip/knee)	(1) Tying a shoe	(1) Conversation. Subject discusses things that excite him or her.
(3.4) Finger tapping (right and left)	(2) Writing a sentence	(2) Picture description. Subject discusses everything he or she sees in a provided picture.
(3.5) Hand movements (flexion/extension)	(3) Writing cursive	(3) Reverse counting. Subject counts backwards from 405 to 375 by 3's.
(3.6) Pronation-supination movements of hand	(4) Fold a piece of paper	(4) Reading. Subject reads an excerpt from a book.
(3.7) Toe tapping	(5) Put on and remove jewelry	(5) Syllables. Subject repeats the word 'PATAKA' as many times as they can in 10 seconds.
(3.8) Leg agility	(6) Use a remote control (press 507, 169, 746)	
(3.9) Arising from chair (arms crossed and fast)	(7) Shake a bottle 5x, open bottle, drink, and close	
(3.10) Gait: 2.5m and 10m	(8) Pour a cup of water, take a drink, return cup to table, take another drink	
(3.12) Postural stability	(9) Eat with a spoon 2x	
(3.13) Posture (eyes open/closed)	(10) Take a lab coat off a hook/table, put it on, button all the buttons, then unbutton all the buttons, take off the lab coat and place back on hook/table	
(3.15) Postural tremor of the hands	(11) Take a sweatshirt off a hook/table, put it on, zip up the zipper, then unzip the sweatshirt, take off the sweatshirt and place back on hook/table	

(3.16) Kinetic tremor of the hands (finger to nose)	(12) Open and close a door	
(3.17) Rest tremor	(13) Carry a book out and back 10m and place on table	
	(14) Carry a suitcase out and back 10m, then hold suitcase for 90 seconds with forearm at 90 degrees.	

Supplementary Table 3. Features extracted for training the tremor and gait classifiers. For each classifier, a checkmark (✓) indicates that the feature was extracted and an asterisk (*) indicates that the feature survived after the feature selection step.

Feature	Description	Tremor Classifier	Gait Classifier
Root mean square value	Root mean square value of a sensor data in a given time window. The RMS value is a measure of signal energy. Value of signal energy feature for accelerometer data is correlated with amount and intensity of motion.	✓*	✓*
Signal range	Range of signal values. Signal range provides a measure of the extremes of motion observed in a given time window of sensor data. Higher range would indicate occurrence of a large excursion in sensor values.	✓*	✓*
Signal entropy	Signal entropy is calculated by estimating Shannon entropy of the probability mass function of a signal[32]. Signal entropy values close to zero indicate that the signal is periodic and smooth, whereas large negative values indicate that the signal is irregular and non-periodic.	✓*	✓*
Correlation coefficient	Cross-correlation coefficient of data from 2 sensor streams (e.g. X axis and Y axis). The cross-correlation coefficient captures degree of co-ordination in the motion between orthogonal directions. Higher values		✓*

	indicate synchronous changes occurring between the two input signals.		
IQR of auto-covariance	Interquartile range of auto-covariance is a measure of long-range dependency or periodicity of a signal. Range of auto-covariance captures if the signal is periodic or irregular.		✓
Mean cross rate	Number of times signal changed from positive to negative normalized by total signal length.		✓*
Range count percentage	Counts of observed time signal is between a given range (percentage).		✓
Dominant frequency	Value of the frequency with the highest magnitude in the normalized power spectrum of the accelerometer signal. Dominant frequency value captures the fundamental frequency of the underlying movement (e.g. tremor, walking) producing the acceleration signal.	✓*	✓*
Dominant frequency magnitude	Magnitude of the dominant frequency in the normalized power spectrum. This feature captures the percentage of total signal energy in the dominant frequency. High values indicate that most of the signal energy is concentrated in the dominant frequency and low values indicate the signal energy is	✓	✓*

	spread across the different frequencies in the power spectrum.		
Ratio of dominant frequency band to total energy in spectrum	Ratio of the energy in the dominant frequency component to the sum of energy in the entire frequency spectrum of a signal. This feature captures periodicity of a signal by calculating the ratio of the energy in the dominant frequency component to the sum of energy in the entire frequency spectrum of a signal.	✓	✓
Spectral flatness	Spectral flatness is calculated by dividing the geometric mean of the power spectrum with the arithmetic mean. Spectral flatness captures the amount of modulation or the level of consistency and ranges from 0 to 1.	✓*	✓*
Spectral entropy[33]	Spectral entropy is calculated by estimating Shannon entropy of the probability mass function of the power spectrum of a signal. Values closer to 1 indicate presence of white noise. Values closer to 0 indicate presence of periodicity in the signal.	✓*	✓*
